# Telomere length was associated with grade and pathological features of meningioma

**DOI:** 10.1038/s41598-022-10157-4

**Published:** 2022-04-12

**Authors:** Keiko Yamakawa, Yuri Mukai, Juanjuan Ye, Mariko Muto-Ishizuka, Masumi Ito, Misa Tanimoto, Futoshi Suizu, Kenichiro Asano, Akira Kurose, Yoko Matsuda

**Affiliations:** 1 Oncology Pathology, Department of Pathology and Host-Defense, Faculty of Medicine, Kagawa University, 1750-1 Ikenobe, Miki-cho, Kita-gun, Kagawa, 761-0793 Japan; 2 Department of Neurosurgery, Hirosaki University Graduate School of Medicine, Hirosaki, Japan; 3 Department of Anatomic Pathology, Hirosaki University Graduate School of Medicine, 5 Zaifu, Hirosaki, 036-8562 Japan

**Keywords:** Cancer, Biomarkers, Oncology

## Abstract

Telomeres are tandem repeats of the TTAGGG sequence at chromosomal ends and afford protection against chromosomal instability. To investigate the contribution of telomere dysfunction in meningiomas, here we estimate the associations between telomere length, tumor grade, and proliferation index in a series of 14 archived samples, using quantitative-fluorescence in situ hybridization, Ki67 immunostaining, and pathological analysis. The number of mitoses per 10 high-power fields (HPF) and Ki67 index was higher in grade III cases than in grade I or grade II cases. Telomere length was negatively associated with both the number of mitoses/10HPF and Ki67 index. Meningioma cases with atypical mitosis, a morphological marker of chromosomal instability, exhibited shortened telomeres. Among telomere-shortened meningioma cases, 40% were grade I, 20% were grade II, and 100% were grade III. In grade I or II meningiomas, shortened telomeres lacked high proliferation activity and atypical mitosis. In conclusion, telomere shortening might be pivotal in the development of high-grade meningioma. Analysis of telomere length might be a selective marker for meningiomas with high-grade malignant potential.

## Introduction

Meningiomas are the most commonly reported brain tumors. Approximately 90% of all meningiomas are slow-growing benign World Health Organization (WHO) grade I lesions, while WHO grade II and III meningiomas are classified as atypical (5–15%) and malignant (1%–3%), respectively^1^. In addition, nearly 10% of meningiomas grow rapidly and are associated with a poor prognosis. Monosomy 22 and inactivating mutations of *NF2* are well-known genetic alterations in meningiomas. In addition, next-generation sequencing recently identified mutations in *TRAF7, AKT1, KLF4, SMO, POLR2A,* and *PIK3C*^2,3^.

Telomeres are tandem repeats of the TTAGGG sequence at chromosomal ends in eukaryotes and play a key role in preventing chromosomal instability^4–6^. A transient period of telomere shortening, and dysfunction drives cancer initiation by inducing chromosomal instability^7,8^. The permanent proliferation of cancer cells depends on the maintenance of telomere length^9^. Telomerase-mediated preservation of telomere function reportedly promotes the development of advanced malignant tumors.

Previous reports have revealed that human telomerase reverse transcriptase (hTERT) promoter mutations C228T and C250T, found in 5.5% of meningiomas, can be associated with poor prognosis and expression of hTERT mRNA, but not telomere length^10^. Chen et al. have reported that Elongated telomere length was measured in 6 of 13 (46.1%) patients with malignant or atypical meningiomas and only 1 of 48 (2.1%) in those with benign tumors (P = 0.0002). The percentage of malignant or atypical meningiomas with detectable telomerase activity or elongated telomere were significantly higher (76.9%) than that of benign tumors (4.0%). The association between telomere length and clinicopathological characteristics of meningiomas remains unclear. Furthermore, most previous studies measuring telomere length have been analyzed by Southern blotting, measuring telomere length in a mixture of cancer and stromal cells. In meningiomas, accurate alterations in telomere length are yet to be clarified. In the present study, using quantitative-fluorescence in situ hybridization (Q-FISH)^11^, telomere lengths were estimated using WHO grade I, II, and III meningiomas. We aimed to clarify the association between telomere length and the WHO grade, and proliferation activity to reveal telomere dysfunction in meningiomas.

## Results

### Telomeres in meningiomas

**Figure 1:**
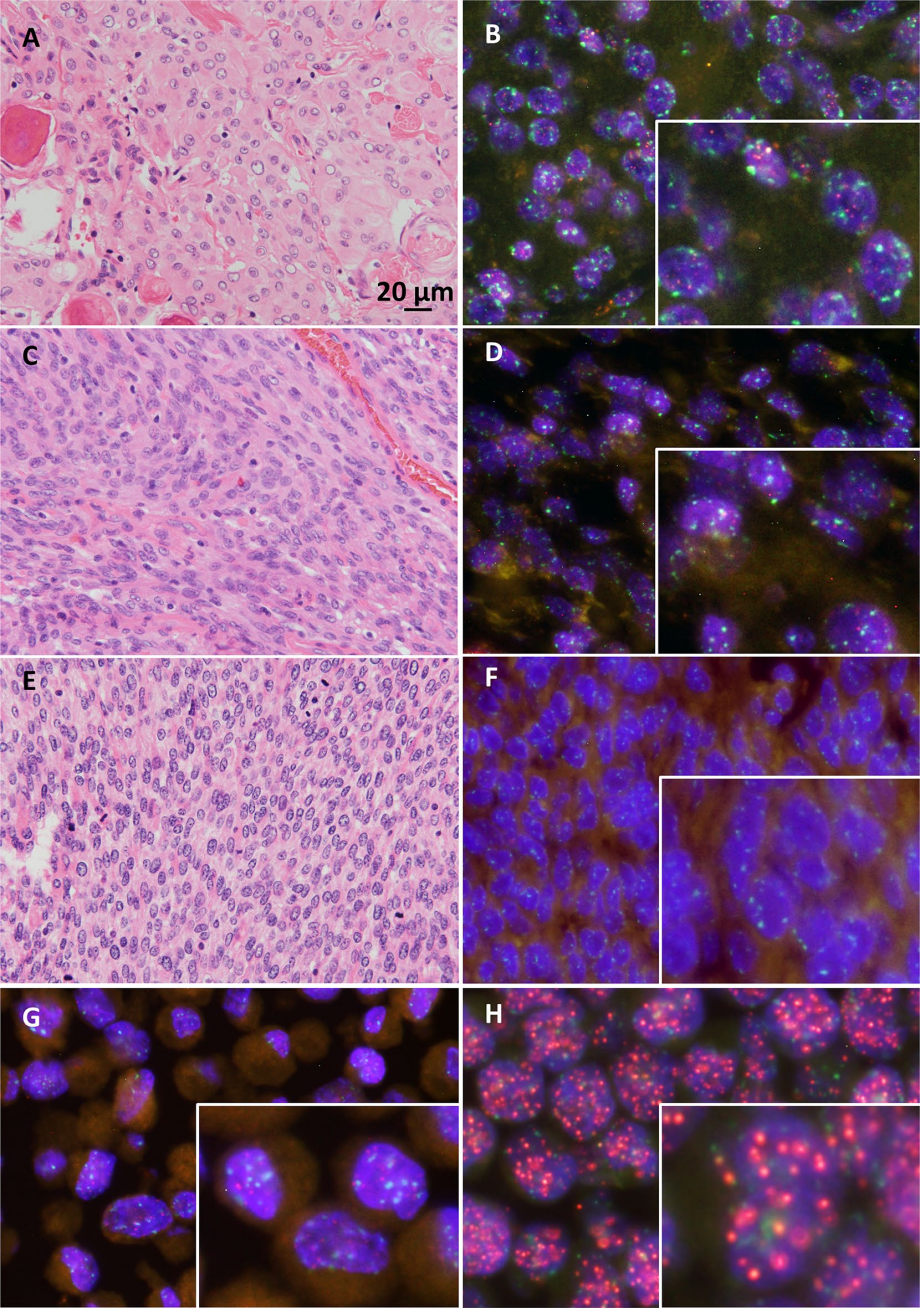
Meningioma cases. (**A**,**B**) WHO grade I; (**C**,**D**) grade II; (**E**,**F**) grade III. (**G**) HFL-1, internal control cells for FISH analysis; (**H**) 1301 cells with long telomeres. (**A**,**C**,**E**) H&E, original magnification × 400; (**B**,**D**,**F**–**H**) FISH images; red, telomere; green, centromere; blue, DAPI; original magnification × 800. *H&E* hematoxylin–eosin; *FISH* fluorescence in situ hybridization, *WHO* World Health Organization.

**Figure 2:**
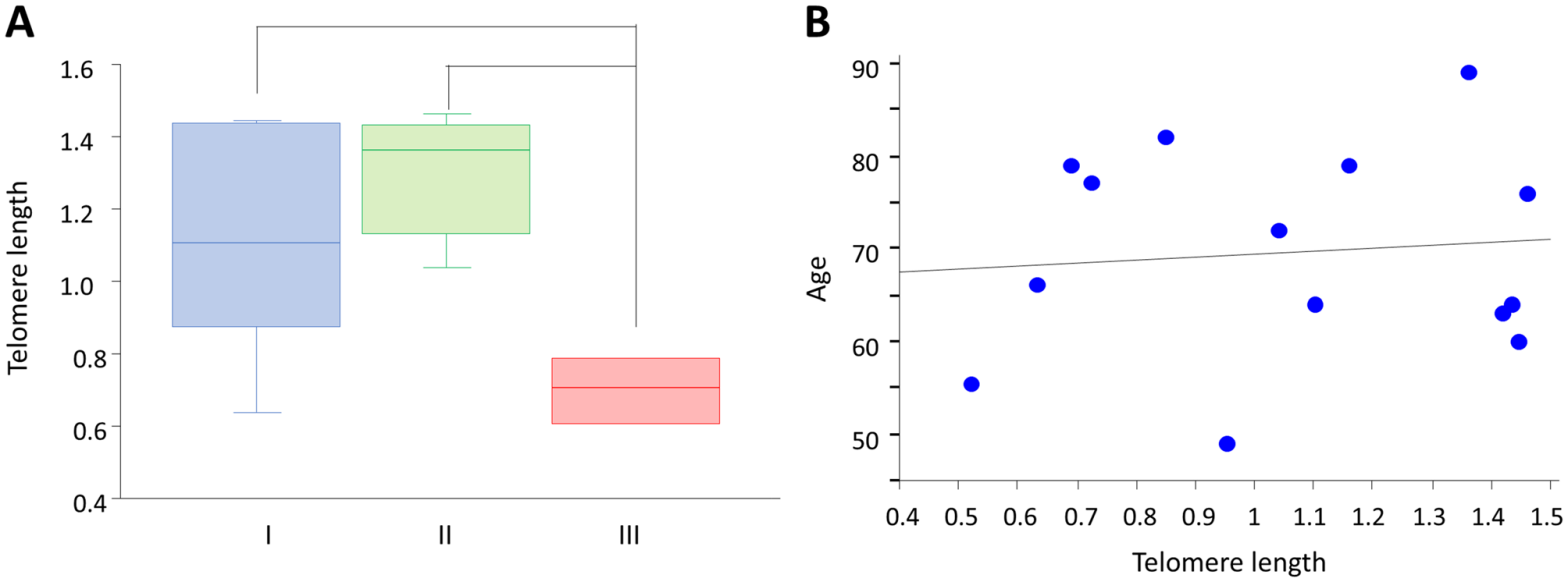
Telomere length was correlated to grade. (**A**) Telomere length and WHO grade. Tukey–Kramer test. (**B**) Telomere length and age. *WHO* World Health Organization.

Based on FISH images, WHO grade I, II, and III meningioma cells exhibited various levels of red signals (red indicates telomeres; Fig. 1, Supplementary Figs. S1, S2 and S3). Red signals in nuclei of grade I and II cases were notable, while grade III cases showed almost no red signals. HFL-1, human fibroblast cells were used for internal control of FISH analysis (Fig. 1G). Human leukemia cell line 1301^12^, which possess long telomere length, represented strong red signals (Fig. 1H). The normalized telomere length, as determined by Q-FISH, was significantly lower in grade III cases than in grade I and II cases (P < 0.01; Fig. 2A), indicating telomere shortening in high-grade meningioma. The telomere length of meningioma cells was not associated with patient age (Fig. 2B).

### Proliferation and telomeres

**Figure 3:**
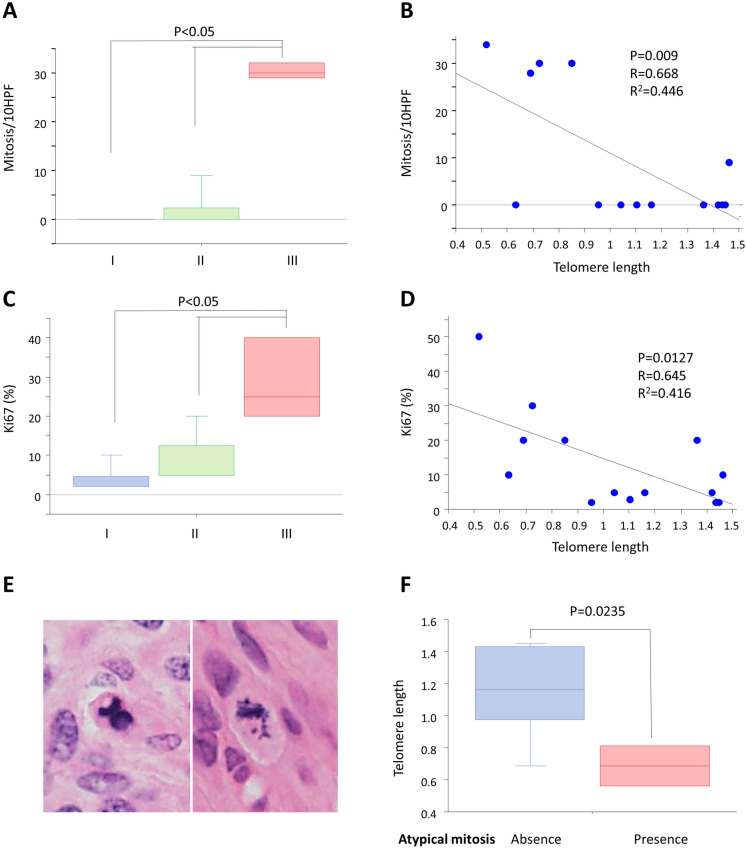
Telomere length was associated with cell proliferation and atypical mitosis. (**A**) WHO grade and the number of mitosis/10HPF. Tukey–Kramer test. (**B**) Telomere length and the number of mitosis/10HPF. (**C**) WHO grade and MIB1 index. Tukey–Kramer test. (**D**) Telomere length and MIB1 index. (**E**) Atypical mitosis in grade III meningiomas. (**F**) Telomere length and atypical mitosis. Student t-test. *HPF* high-power fields, *WHO* World Health Organization.

Mitosis is associated with the prognosis of meningiomas^13^ and is a quantitative criterion used in meningioma grading^14^. The number of mitosis/10 high-power fields (HPF; 0.24 mm^2^) HPF was higher in grade III cases than in grade I or grade II cases (P < 0.05, respectively; Fig. 3A). In addition, the normalized telomere length was negatively associated with the number of mitosis/10HPF (P = 0.009; R = 0.668; R^2^ = 0.446; Fig. 3B). The MIB1 index revealed similar results to mitosis; grade III cases showed a higher MIB1 index than grade I or II cases (P < 0.05, respectively; Fig. 3C), and the normalized telomere length was negatively associated with the MIB1 index (P = 0.0127; R = 0.645; R^2^ = 0.416; Fig. 3D).

Atypical mitosis, such as multipolar, lag-type, ring, and asymmetrical mitosis, including anaphase bridges^15^ (Fig. 3E), is a morphological marker of chromosomal instability^11^. Herein, we observed that the normalized telomere length was lower in cases with atypical mitosis than in cases without atypical mitosis (P = 0.0235; Fig. 3F), suggesting the association of shortened telomeres with chromosomal instability.

Patients were divided into two groups: those with short and long telomeres based on the median value of normalized telomere length (cutoff value, 1.072; Table 1). Telomere length showed a statistically significant association with WHO grade and the number of mitosis/10HPF (0.0498 and 0.0255, respectively). On categorizing telomere-shortened meningioma cases, 40% were grade I, 20% were grade II, and 100% were grade III. In addition, telomere-shortened grade I (n = 1) and grade II (n = 2) cases lacked high proliferation activity and atypical mitosis.

**Table 1:** Clinicopathological characteristics of meningioma with short or long telomere.

	Short	Long	*P* value
Age, med (min–max)	64 (49–84)	72 (60–89)	0.7232
Sex, female/male, n	2/5	2/5	> 0.9999
Location	Convexity 3/Cranial fossa 2/Spura cellular 1/Tetrium cerebelli 1	Front 1/Olfactory groove 1/Parietal 1/Parasagittal 1/Convexity 1/Sellar 1/	0.3253
WHO grade, I/II/III, n	2/1/4	3/4/0	0.0498*
Mitosis/10HPF, med (min–max)	28 (40–34)	0 (0–9)	0.0255*
MIB1 index, med (min–max)	20 (2–50)	5 (2–20)	0.0800
Atypical mitosis, presence/absence	3/4	0/7	0.1927

Patients were divided into two groups according to the median value of normalized telomere length (cutoff value, 1.072).

*HPF* high-power fields, *WHO* World Health Organization.

## Discussion

The present study revealed the following new findings: (1) all grade III meningiomas exhibited telomere shortening and high proliferation activity; (2) 30% of grades I and II meningiomas showed telomere shortening but not high proliferation activity and atypical mitosis, a marker of chromosomal instability. These results indicate that telomere shortening is pivotal for the development of high-grade meningiomas.

Chromosomal instability is characterized by an increase in the rate of addition or deletion of entire chromosomes or chromosomal sections^16^ and many tumors have been shown to exhibit chromosomal abnormalities. It has been reported that meningiomas harbor monosomy 22 as the sole abnormality, and some cases possess a more complex karyotype^17^. In addition, 1p/19q deletion/imbalance^18^ and tetraploidy^19^ have been reported in meningiomas. DNA damage response, telomere shortening, and spindle assembly checkpoint abnormalities have been shown to induce chromosomal instability. Although the present study indicates the association between telomere shortening and chromosomal instability in meningiomas, there has been no report in meningiomas to conclude that telomere shortening is linked to chromosomal instability.

All grade III meningiomas showed telomere shortening and high proliferation activity, while a small number of grades I and II meningiomas revealed telomere shortening but lacked high proliferation activity and atypical mitosis. There is a possibility that grade I and II meningiomas with shortened telomeres lack high proliferation activity and atypical mitosis might indicate a lack of telomerase or gene mutations involving cell proliferation. On obtaining mutations, tumor cells may develop into a grade III meningioma. These results indicate that a small portion of grade I and II meningiomas possess existing telomere abnormalities and thus might progress to grade III meningioma. Accordingly, telomere analysis can potentially identify high-risk patients.

The present study has a limitation. The cohort is quite small to carry out univariate/multivariate analyses necessary to determine the real impact on clinical outcome. Hence, further analysis using a larger cohort is warranted.

In conclusion, grade III meningiomas show telomere shortening and high proliferative activity. Thus, despite the small number of cases assessed, telomere length might be a selective marker for meningiomas with high-grade malignant potential.

## Materials and methods

### Patients

Surgically resected tissues were obtained from 14 patients who underwent surgical intervention at the Hirosaki University Hospital between 2014 and 2020. Written informed consent to use the tissue samples was obtained from all patients prior to the study. The study was conducted in accordance with the principles of the Declaration of Helsinki (2013). All experiments were approved by the ethics committee of Hirosaki University Hospital (No. 2021–101) and Kagawa University (2019-209).

### Tissue processing and pathological assessments

Formalin-fixed and paraffin-embedded tissues from advanced tumor sites were used. The tissues were serially sliced into sections (3-μm thick) for hematoxylin–eosin (H&E) staining, immunostaining, and FISH. Pathological specimens were examined by pathologists based on the WHO Classification of Tumors of the Central Nervous System^1^. Mitotic counts in 10HPF were obtained using H&E-stained specimens at a magnification of × 400. The presence or absence of atypical mitosis, including multipolar, lag-type, ring, and asymmetrical mitosis, as well as anaphase bridges, was determined using H&E-stained specimens at 1000 tumor cells and a magnification of × 400, as previously reported^15^. In addition, Ki67 (MIB-1; Dako, Glostrup, Denmark) immunostaining was performed to evaluate proliferation activity.

### Quantitative fluorescence in situ hybridization for the analysis of telomere length

The slides were processed using FISH, as previously reported^11^. In brief, tissue sections were hybridized with 200 nM PNA probes for the telomere (telo C-Cy3 probe, 5-CCCTAACCCTAACCCTAA-3; Panagene, Daejeon, Korea) and centromere (Cenp1-FITC probe, 5-CTTCGTTGGAAACGGGGT-3; Panagene) at 80 °C for 3 min, followed by 1 h at room temperature. Then, nuclei were stained with DAPI (Molecular Probes, Eugene, OR, USA). FISH images were captured using a fluorescent microscope (FSX100; Olympus, Tokyo, Japan) at × 800 magnification.

Image J (version 1.53a, Wayne Rasband, National Institutes of Health; modified by the plug-in AsKey, Kagawa, Japan) was used to estimate the red, green, and blue intensities of individual nuclei. As an entire nucleus will not necessarily be captured within any given tissue section, the total corrected telomere signal for each nucleus was normalized by the corresponding integrated optical density of the centromere as the telomere/centromere ratio. Over 100 cells were analyzed for each sample. As a control to account for variations in sample preparation, FISH was also performed on block sections of a cultured cell strain, HFL-1 (Fig. 1G with a population doubling level of 20). The normalized telomere length for each case was calculated as follows: [median value of telomere/centromere ratio of target cells]/[median value of telomere/centromere ratio of control HFL-1 cells].

### Statistical analysis

Comparison of two groups was performed using the unpaired *t*-test, Mann–Whitney U-test, χ^2^ test, and Fisher’s exact test. Three or more groups were compared using analysis of variance (ANOVA) and Tukey’s test. Correlations were assessed using Pearson’s and Spearman’s correlation coefficients. Statistical analyses were performed using JMP Pro 14 (SAS Institute Inc., Cary, NC, USA).

## Supplementary Information

Supplementary Figure S1.

Supplementary Figure S2.

Supplementary Figure S3.

Supplementary Legends.

## Data Availability

All relevant data are available within the article and Supplementary files, or available from the authors upon request.
