# Supplementary figures and images for: Telomere length was associated with grade and pathological features of meningioma
Source: Sci Rep. 2022 Apr 12;12:6143. doi: 10.1038/s41598-022-10157-4 (PMC9005517; doi:10.1038/s41598-022-10157-4)

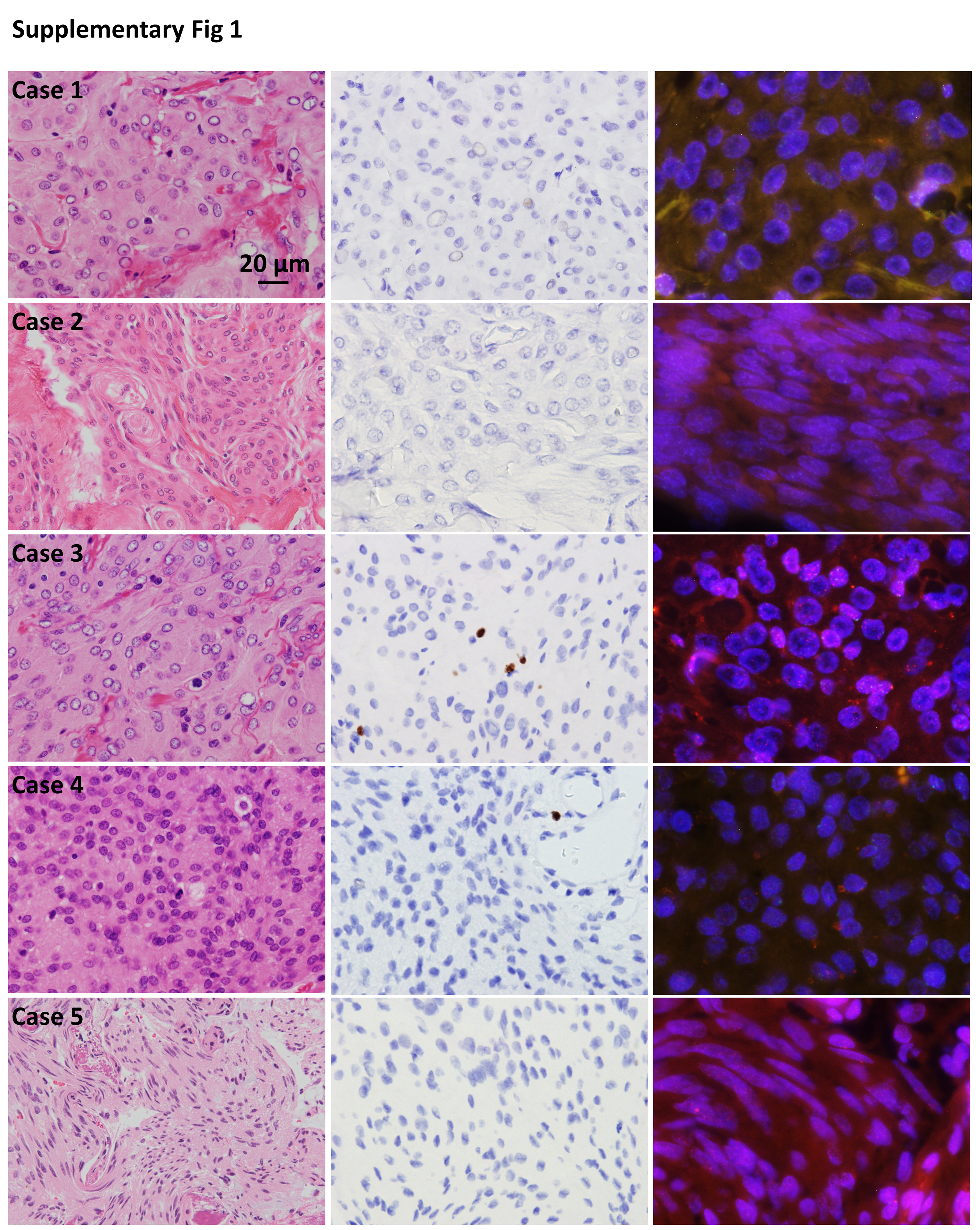

Supplement: Supplementary file 1 — Supplementary Figure S1. [file 41598_2022_10157_MOESM1_ESM.tif]

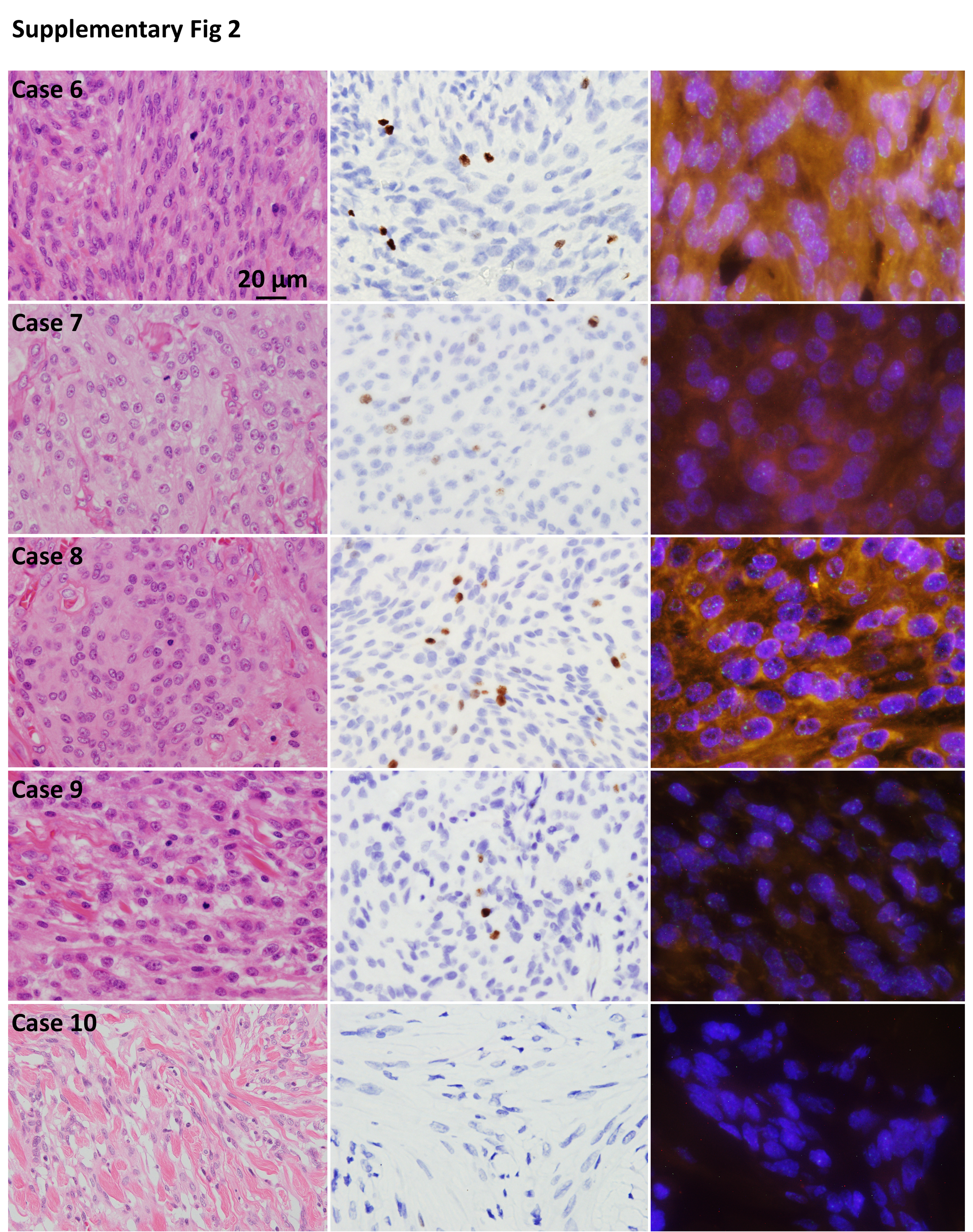

Supplement: Supplementary file 2 — Supplementary Figure S2. [file 41598_2022_10157_MOESM2_ESM.tif]

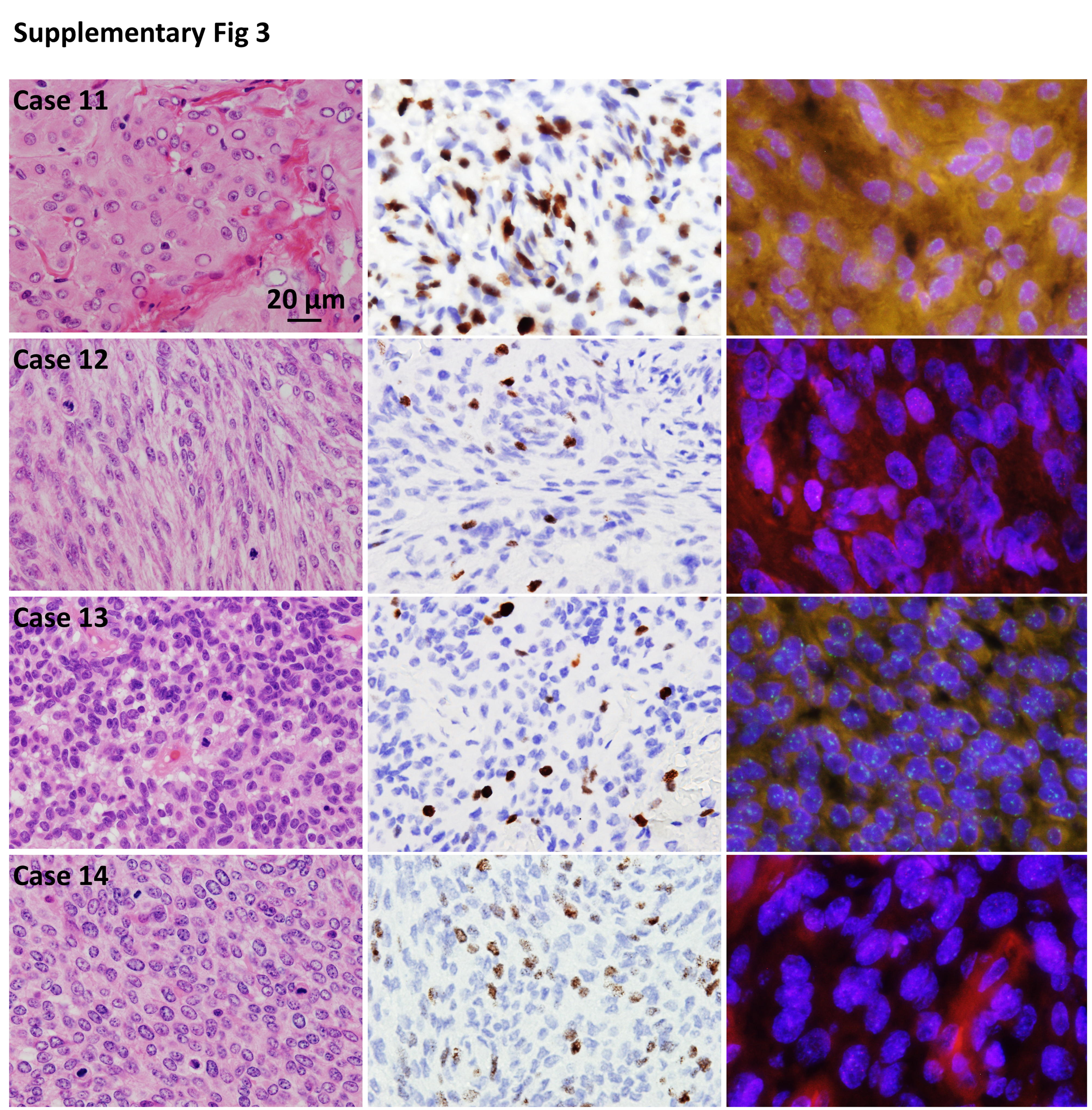

Supplement: Supplementary file 3 — Supplementary Figure S3. [file 41598_2022_10157_MOESM3_ESM.tif]
