# Supplementary material for: Telomere length was associated with grade and pathological features of meningioma
Source: Sci Rep. 2022 Apr 12;12:6143. doi: 10.1038/s41598-022-10157-4 (PMC9005517; doi:10.1038/s41598-022-10157-4)
Supplement: Supplementary file 4 — Supplementary Legends. [file 41598_2022_10157_MOESM4_ESM.docx]

**Legends for supplementary figures**

**Supplementary Fig. 1. WHO Grade I Meningioma cases.** Left panels, H&E; center panels, immunohistochemical staining for Ki67, original magnification ×400; right panels, FISH images; red, telomere; green, centromere; blue, DAPI; original magnification ×800.

**Supplementary Fig. 2. WHO Grade II Meningioma cases.** Left panels, H&E; center panels, immunohistochemical staining for Ki67, original magnification ×400; right panels, FISH images; red, telomere; green, centromere; blue, DAPI; original magnification ×800.

**Supplementary Fig. 3. WHO Grade III Meningioma cases.** Left panels, H&E; center panels, immunohistochemical staining for Ki67, original magnification ×400; right panels, FISH images; red, telomere; green, centromere; blue, DAPI; original magnification ×800.
